# Vibration Control and Micro-Forming Quality Guarantee of BMF-Based UHPC Wet Joints Under Traffic Loads Using Tuned Mass Dampers

**DOI:** 10.3390/ma19081564

**Published:** 2026-04-14

**Authors:** Zhenwei Wang, Lingkai Zhang, Chujia Zhou, Peng Wang

**Affiliations:** 1 Zhejiang Highway and Water Transportation Engineering Consulting Group Co., Ltd., Hangzhou 310011, China; zhanglk1994@163.com; 2 Zhejiang Institute of Communications Co., Ltd., Hangzhou 310013, China; zhouchujia@163.com; 3 Guangdong Provincial Key Lab of Durability for Marine Civil Engineering, Shenzhen University, Shenzhen 518060, China; pwangal@connect.ust.hk

**Keywords:** basalt micro fiber (BMF), ultra-high-performance concrete (UHPC), bridge widening, interfacial transition zone (ITZ), vehicle-bridge coupled vibration, tuned mass damper (TMD), early-age forming quality

## Abstract

In bridge widening projects under uninterrupted traffic conditions, vehicular vibration easily leads to damage in the interfacial transition zone (ITZ) and microstructural degradation of early-age concrete in wet joints. Taking a typical hollow slab-low T-beam widening structure as the object, this study introduces basalt micro fiber (BMF)-based ultra-high-performance concrete (UHPC) as the wet joint material and establishes a refined vehicle–bridge coupled dynamic model considering the time-varying stiffness of the joint material and road roughness excitation. The research indicates that although UHPC possesses excellent ultimate mechanical properties, its early-age setting process is extremely sensitive to vehicle-induced vibration. Numerical analysis reveals that while traditional temporary steel fixtures can effectively control the vertical relative displacement between the new and old girders within the critical value of 5.5 mm, the peak particle velocity (PPV) induced by heavy vehicles (buses and trucks) during the early pouring stage (<12 h) significantly exceeds the safety threshold of 3 mm/s, posing a severe threat to the directional distribution of steel fibers and interfacial bond strength. Therefore, this paper designs a single tuned mass damper (TMD) optimized based on Den Hartog’s fixed-point theory. Simulation results confirm that with the TMD configured, the vibration responses induced by buses across the entire speed range (≤120 km/h) are reduced below the safety limit; the vibration velocity induced by heavy trucks is also effectively controlled when combined with an 80 km/h speed limit. The collaborative strategy of “passive TMD vibration reduction + active traffic speed limit” proposed in this paper provides a theoretical basis for guaranteeing the early-age micro-forming quality of UHPC wet joints and overall traffic efficiency.

## 1. Introduction

### 1.1. Research Background

With the continuous economic development in China, existing expressways are increasingly unable to meet the growing traffic demands. Concurrently, as traffic corridor resources between urban agglomerations become saturated, the expansion and capacity enhancement of existing highways have become crucial for alleviating traffic pressure. According to the 14th Five-Year Plan for the National Highway Network (July 2022) [1], China aims to establish a national highway network of approximately 461,000 km by 2035. This plan includes about 58,000 km of new and upgraded expressways, with capacity expansion projects constituting a significant 51.7% (approximately 30,000 km). This highlights that the reconstruction and widening of expressways will be a core task in future transportation infrastructure development.

In such reconstruction and expansion projects, bridge widening is a critical determinant of success. However, bridges requiring widening are often situated on heavily trafficked routes. Complete traffic closure would not only cause severe congestion but also lead to significant economic and social impacts [2]. Consequently, achieving a reliable structural connection between existing and new bridge sections under uninterrupted traffic conditions has become a key technical challenge. Based on this context, the present study focuses on bridge widening under uninterrupted traffic, employing numerical simulations to analyze the impact mechanism of traffic-induced vibrations on the mechanical properties of wet-joint concrete. Corresponding vibration control and optimization strategies are proposed to provide a theoretical basis and technical support for similar engineering projects.

### 1.2. Field Survey of Widened Bridges

A project in Zhejiang Province involved widening an expressway to an eight-lane standard with a design speed of 120 km/h. The roadbed widths were 42.0 m for integral sections and 20.75 m for separated sections. The design live load for new structures was Highway-I class, while existing structures maintained their original design standards of Vehicle-Super 20 and Trailer-120. Other technical specifications adhered to the Technical Standard of Highway Engineering (JTG B01-2003).

The project involved 77 widened bridges. At the handover inspection in July 2017, 31 bridges exhibited defects such as cracking, water seepage, and efflorescence, with transverse cracking being the most prevalent issue. The number of defects at this stage significantly exceeded those found at the final completion inspection. This discrepancy, especially considering that traffic was maintained during construction, suggests that traffic-induced vibrations and differential deflections likely had adverse effects on the concrete wet joints, reflecting significant construction quality issues. After repairs, the bridges were opened to traffic in September 2018 for a trial operation. However, at the completion inspection in March 2022, 15 bridges still showed transverse cracking, affecting over half of their spans, with up to 20 cracks in a single span and a maximum width of 0.2 mm.

The persistence of these defects at the final inspection indicates that the long-term effects of operational traffic vibrations on widened bridges cannot be overlooked. Therefore, this study posits that a thorough investigation into the dynamic effects of traffic vibrations is necessary to comprehensively evaluate the performance of widened bridges and guide future improvements.

### 1.3. Literature Review

A substantial body of academic research has investigated the detrimental effects of traffic-induced vibration on early-age concrete. Harsh et al. [3] reported that continuous vibration during the initial curing period could reduce the compressive strength of plastic concrete by approximately 10% and the bond strength between reinforcement and the concrete by 5.9%. Conversely, for concrete with a slump of less than 75 mm, performance degradation under traffic conditions was found to be insignificant. Zhang et al. [4] (2015) identified a “disturbance-sensitive period” for early-age concrete, defined by a penetration resistance between 3.5 and 28 MPa. During this stage, vehicle–bridge coupled vibration can cause severe damage; however, the incorporation of crack-resistant fibers and self-healing admixtures has been proven to significantly mitigate these effects. Ansell and Silfwerbrand [5] (2003) conducted a comprehensive literature review and identified a “vulnerable window” (approximately 3 to 12 h after casting) during which early-age concrete is highly sensitive to vibration. Accordingly, they proposed age-based limits for peak particle velocity (PPV), recommending a strict threshold of 35 mm/s during this critical phase to prevent internal damage. Recently, Qiao et al. [6] (2025) investigated the bond-slip behavior between newly cast concrete and steel rebars under traffic-induced vibration during bridge widening via central pull-out tests, revealing that high-frequency vibration significantly weakens the bond stress and increases the slip.

Research on bridge widening under uninterrupted traffic primarily focuses on two directions: enhancing the mechanical properties of joint materials under vibration and optimizing construction methods to mitigate vibration at the source. Regarding material performance, Chen [7] compared the disturbance resistance of normal concrete (NC) and ultra-high-performance concrete (UHPC). Zeng et al. [8] (2023) developed normal and high-performance concretes resistant to differential disturbance, noting that fiber reorientation leads to fluctuations in tensile strength. Liu et al. [9] (2024) revealed a dual mechanism wherein minor vibrations enhance strength through a densification effect, whereas excessive vibrations impair interfacial bonding. Given its exceptional mechanical properties and interfacial bonding capabilities, UHPC has emerged as an ideal material for bridge splicing.

However, recent micro-mechanical studies on UHPC indicate that it is extremely sensitive to vehicle-induced vibrations at early ages, particularly from the initial to final setting stages. At the macroscopic response level, Zhao et al. [10] (2025) found that high-amplitude vibrations cause cracks at UHPC joints, reducing the ultimate bearing capacity by 19% compared to non-vibrated specimens. At the micro-mechanistic level, Leng et al. [11] revealed that high-energy vibrations disrupt the bonding and friction between aggregates and the matrix, causing the interfacial transition zone (ITZ) to degrade from a “dense framework” to a “cracked framework” containing microcracks. Research on lightweight UHPC by Liu et al. [12] (2025) further confirmed that vibration during the initial to final setting period severely hinders the accumulation of hydration products around steel fibers, leading to a surge in ITZ porosity and a sharp decline in the interfacial bond strength between the fibers and the matrix. Furthermore, a mesoscopic mechanistic study by Yang et al. [2] (2024) demonstrated that low-amplitude minor vibrations (e.g., 1–3 mm) facilitate better directional alignment of steel fibers along the tensile direction, thereby improving flexural strength; conversely, violent vibrations with large amplitudes disrupt fiber orientation, resulting in a drastic 23.9% decrease in flexural strength. A comprehensive study by Basler et al. [13] (2023) on the effects of sinusoidal vibration on early-age concrete also emphasized the complex coupling effects among various vibration parameters and highlighted the necessity of controlling early-age vibration energy (e.g., PPV).

In terms of vibration control, some scholars have primarily focused on traffic management. For instance, Zhou [14] successfully controlled the PPV at wet joints within a safe range by implementing weight limits and lane closures. Kwan and Ng [15] (2006) systematically evaluated four mitigation measures, demonstrating that temporary rigid shear connections are the most effective in achieving synchronous deformation. In practical engineering, such as the widening project of the Hang-Jin-Qu Expressway, a common technique involves spanning new and old girders with steel fixtures (e.g., I22 I-beams) [16]. Although this method ensures construction safety by maintaining a low-deformation state during concrete curing, high-frequency dynamic vibrations during the operational phase may still induce microcracks, thereby potentially compromising long-term durability.

In summary, a critical research gap remains: while existing temporary rigid connections can restrict macroscopic displacement, they fail to mitigate high-frequency peak particle velocity (PPV) under operational traffic. Consequently, excessive PPV during the construction phase can irreversibly damage the early-age microstructural formation of UHPC (e.g., ITZ degradation and fiber disorientation), despite its high post-hardening strength. To bridge this gap, this study proposes an innovative collaborative vibration control strategy. The core innovation lies in strictly controlling the PPV at the wet joints by integrating a passive Tuned Mass Damper (TMD) optimized via Den Hartog’s theory with active traffic speed limits. By establishing a refined vehicle–bridge coupled dynamic model, this research systematically evaluates the dynamic response of BMF-based UHPC wet joints, thereby fundamentally guaranteeing their early-age interfacial forming quality and long-term durability under uninterrupted traffic conditions.

## 2. Theoretical and Numerical Analysis Methods and Their Validation

To ensure the accuracy and reliability of the vehicle–bridge interaction analysis model, this study employs two independent methods for modeling and solving: a theoretical calculation based on the mode superposition method and a numerical simulation using the finite element software ANSYS (Release 2021 R1) [17]. Both methods are applied to the same simply supported beam–moving spring-mass system. By comparing their dynamic response time histories, the validity of the finite element model is verified. This chapter systematically elaborates on the principles, modeling processes, and comparative analysis of the results from both methods, thereby demonstrating the reliability of the constructed finite element model for handling vehicle–bridge interaction problems and laying a solid foundation for subsequent complex case studies.

### 2.1. Theoretical Calculation Model Based on Mode Superposition

The core idea of the theoretical calculation is to discretize the continuous vibration problem of the beam into a finite number of modal coordinates and obtain the system’s dynamic response by solving a set of ordinary differential equations. This model utilizes the mode superposition method combined with the Newmark-β numerical integration method for time–history analysis.

#### 2.1.1. Model Formulation and Equations of Motion

The transverse displacement of the simply supported beam can be expressed as:

(1) $$y\left(x,t\right)=\sum_{i=1}^{n}\phi_{i}\left(x\right)\eta_{i}\left(t\right)$$

where $\phi_{i}\left(x\right)=\sin\left(\frac{i\pi x}{L}\right)$ is the *i*-th mode shape function, *L* is the beam span, and $\eta_{i}\left(t\right)$ is the *i*-th modal coordinate. This study considers the first n=10 modes.

The vehicle is simplified as a two-degree-of-freedom spring-mass-damper system, consisting of an unsprung mass $m_{1}$ and a sprung mass $m_{2}$, which are connected by a spring $k_{1}$ and a damper $c_{1}$. The vehicle travels from the left end of the beam to the right at a constant speed *v*, with its position given by $x_{v}=vt$. The interaction force between the vehicle and the bridge is composed of gravitational, spring, and damping forces.

Considering the vehicle–bridge interaction, the system’s equation of motion can be written as:

(2) $$\left[M\left(t\right)\right]\left\{\ddot{\eta }\left(t\right)\right\}+\left[C\left(t\right)\right]\left\{\dot{\eta }\left(t\right)\right\}+\left[K\left(t\right)\right]\left\{\eta \left(t\right)\right\}=\left\{F\left(t\right)\right\}$$

where [M(t)], [C(t)], and [K(t)] are the time-varying mass, damping, and stiffness matrices of the system, respectively. These matrices include the inherent properties of the beam as well as the coupling terms that change with the vehicle’s position. F(t) is the time-varying load vector. The matrix parameters are updated in real-time according to the instantaneous position of the vehicle to ensure the accuracy of the dynamic response.

#### 2.1.2. Numerical Solution Method

As the aforementioned equations of motion constitute a time-varying system, a direct analytical solution is difficult to obtain. Therefore, the Newmark-β method is employed for time–history integration. This method is a widely used implicit integration scheme known for its unconditional stability and high computational accuracy, making it well-suited for structural dynamics problems. The main steps are as follows:

1. **Define Newmark-β parameters.** In this study, we take $\gamma =0.005,\alpha ={\left(1+\gamma \right)}^{2}/4=0.255025,\delta =0.5+\gamma =0.505$;
2. **For each time step Δt**, construct the effective stiffness matrix $\left[K_{\mathrm{eff}}\right]$ and the effective load vector $\left\{F_{\mathrm{eff}}\right\}$ based on the displacement, velocity, and acceleration from the previous time step, as well as the current load;
3. **Solve the system of linear equations:**
  (3) $$\left[K_{\mathrm{eff}}\right]\left\{\Delta \eta \right\}=\left\{F_{\mathrm{eff}}\right\}$$
  to obtain the incremental modal coordinates {Δη};
4. **Update the modal displacements, velocities, and accelerations.** Repeat the iteration until the vehicle has traversed the bridge, finally outputting the time–history curves for displacement and acceleration at the mid-span of the beam and for the unsprung mass.

This method can effectively capture the dynamic characteristics of the vehicle–bridge coupled system, providing reliable benchmark data for the validation of the finite element model.

### 2.2. ANSYS Finite Element Model Based on the Contact Method

Finite Element Analysis (FEA) is a powerful numerical tool that solves partial differential equations by discretizing a continuous body into a finite number of elements and nodes, thereby transforming them into a system of algebraic equations. This study utilizes a contact-based approach in ANSYS to establish a vehicle–bridge interaction model, simulating the vehicle’s movement along the simply supported beam and their mutual interaction.

#### 2.2.1. Model Establishment and Element Selection

- **Beam Model:** The simply supported beam is discretized using BEAM189 elements. This is a three-dimensional two-node beam element suitable for linear, large-rotation, and large-strain analyses, capable of accurately representing the beam’s bending stiffness and mass distribution. The material parameters of the beam are kept consistent with the theoretical model, with an elastic modulus of $E=3.45\times 10^{10}\;\mathrm{Pa}$ and a density of $\rho =2600\;\mathrm{kg}/m^{3}$.
- **Vehicle Model:** The vehicle consists of an unsprung mass $\left(m_{1}\right)$ and a sprung mass $\left(m_{2}\right)$, both modeled as concentrated masses using MASS21 elements. They are connected by a COMBIN14 element, which represents the spring and damping characteristics of the suspension system. The suspension stiffness is set to $k_{1}=3\times 10^{6}\;N/m$ and the damping coefficient is $c_{1}=1\times 10^{4}$ N·s/m, consistent with the theoretical model.

#### 2.2.2. Contact Method and Moving Load Simulation

The accurate simulation of the contact relationship is key to the vehicle–bridge interaction. This study employs contact elements to model the complex nonlinear contact behavior. Specifically, the contact point corresponding to the unsprung mass is described by CONTA175 elements, while the upper surface of the beam serves as the target surface, modeled with TARGE169 elements. By setting the parameter KEYOPT,4,12,2, the contact is configured to allow sliding but prevent separation.

The moving load is implemented in two stages:

1. **Static Analysis:** Gravitational loads, $m_{1}g$ and $m_{2}g$, are applied to the nodes of the unsprung and sprung masses, respectively, to simulate the vehicle’s self-weight.
2. **Transient Analysis:** The analysis type is set to transient (antype,trans). The vehicle nodes are moved along the longitudinal direction of the beam at a constant speed *v* by applying a displacement boundary condition, (d,nmax,ux,ti·v), while maintaining contact with the beam surface.

#### 2.2.3. Analysis Settings

To ensure the accuracy and convergence of the results, the model enables the large deformation effect (nlgeom,on) and uses automatic time sub-stepping (autots,on). During the vehicle’s movement, ANSYS adaptively adjusts the time step size to obtain a stable and precise dynamic response.

### 2.3. Comparison and Validation of the Two Methods

**Figure 1:**
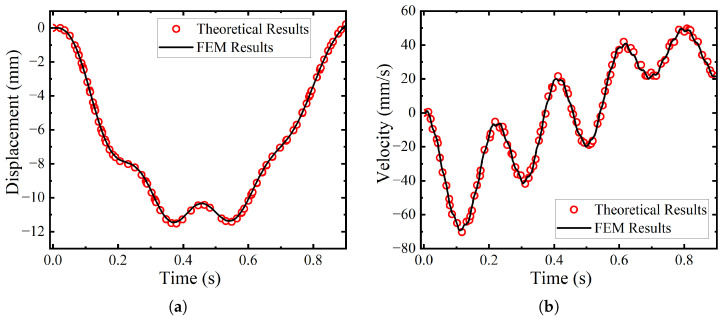

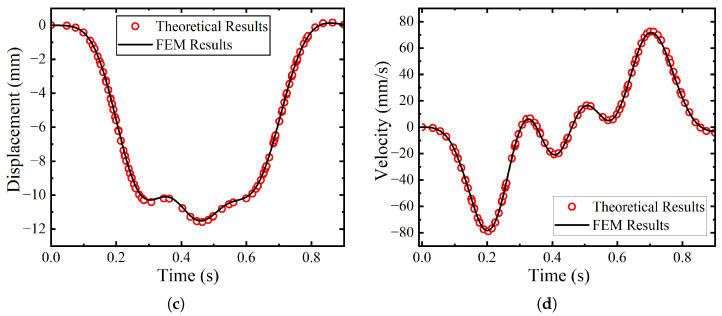
Comparisons of dynamic responses between theoretical results and FEM results. (**a**) Mid-span vertical displacement. (**b**) Mid-span vertical velocity. (**c**) Sprung mass vertical displacement. (**d**) Sprung mass vertical velocity.

To validate the rationality and accuracy of the ANSYS finite element model based on the contact method, these key dynamic responses were compared with the results from the theoretical calculation program (see Figure 1). The results indicate that the time–history curves obtained from both methods exhibit a high degree of consistency in terms of overall trend, vibration amplitude, frequency, and the timing of peak values. Key quantitative metrics, such as peak values, are also in close agreement, with only minor discrepancies arising from differences in solution algorithms and modeling details. This good consistency sufficiently demonstrates that the finite element model can accurately simulate the dynamic response of the vehicle–bridge coupled system, laying a solid foundation for subsequent, more complex parametric studies.

## 3. Establishment of the Finite Element Model

This chapter details the process of establishing the finite element model for the vehicle–bridge dynamic interaction analysis. It covers four main aspects: the selection and establishment of vehicle models, the refined modeling of the bridge superstructure (a widened bridge with hollow-core slabs and shallow T-beams), the mathematical simulation of road surface roughness, and the point-to-surface contact coupling method used to implement the dynamic interaction.

### 3.1. Vehicle Model

**Figure 2:**
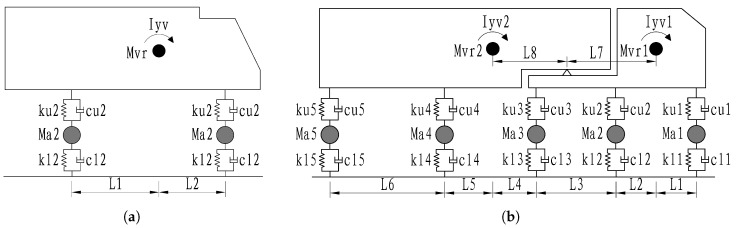
Numerical model of vehicle. (**a**) Two-axle vehicle. (**b**) Five-axle vehicle.

In vehicle–bridge interaction analysis, the selection of the vehicle model is crucial for accurately predicting the dynamic response of the coupled system. To ensure the model comprehensively reflects the diversity and complexity of actual traffic loads, this study selects four typical vehicle types for analysis: a passenger car, a light-duty truck, a bus (all two-axle models), and a heavy-duty truck (a five-axle model) [18]. This diverse selection accounts for significant differences in key parameters such as gross vehicle mass, axle load distribution, and suspension stiffness. A three-dimensional spatial vibration model for each vehicle is constructed using a multi-body dynamics approach, composed primarily of mass, spring, and damper elements (as shown in Figure 2).

Specifically, MASS21 elements are used to simulate concentrated mass points representing the mass and rotational inertia of the vehicle body and wheelsets, while COMBIN14 elements are used to model the spring-damper systems, representing the stiffness and damping characteristics of the suspension and tires. As the mechanical parameters of the vehicle suspension and tires are key factors influencing the vibration response of the vehicle–bridge system, their accurate modeling is essential for realistically capturing the dynamic characteristics. The detailed parameters and dimensions of each vehicle model used in this paper are provided in Table 1. Through a comprehensive analysis of these four representative vehicles, this study can more realistically simulate the coupled vibrations of the bridge under actual traffic loads, providing a reliable scientific basis for bridge structural design, condition assessment, and maintenance decisions.

### 3.2. Bridge Superstructure Model

#### 3.2.1. Background and Model Selection

Prestressed concrete hollow-core slab beams were widely used in the early construction of short-span highway bridges in China. However, long-term operational experience has shown that their hinge-jointed connection method has inherent defects, with joint failure being a primary cause of bridge distress.

To address this widespread issue, the Zhejiang Provincial Department of Transportation has restricted the use of hollow-core slabs where vertical clearance permits, recommending T-beams or small box girders instead [19]. This has led to the common practice of using structurally superior shallow T-beams for the new sections when widening and retrofitting existing hollow-core slab bridges. However, research on the connection of such dissimilar beam types is relatively scarce. Due to the differences in structural stiffness and natural frequencies between the two components, significant differential deflections may arise between the old and new sections after widening, posing a challenge to the long-term performance of the wet joint. In view of this, a typical short-span bridge widened with shallow T-beams connected to existing hollow-core slabs was selected as the object of this study.

#### 3.2.2. Bridge Structure and Dimensions

**Figure 3:**
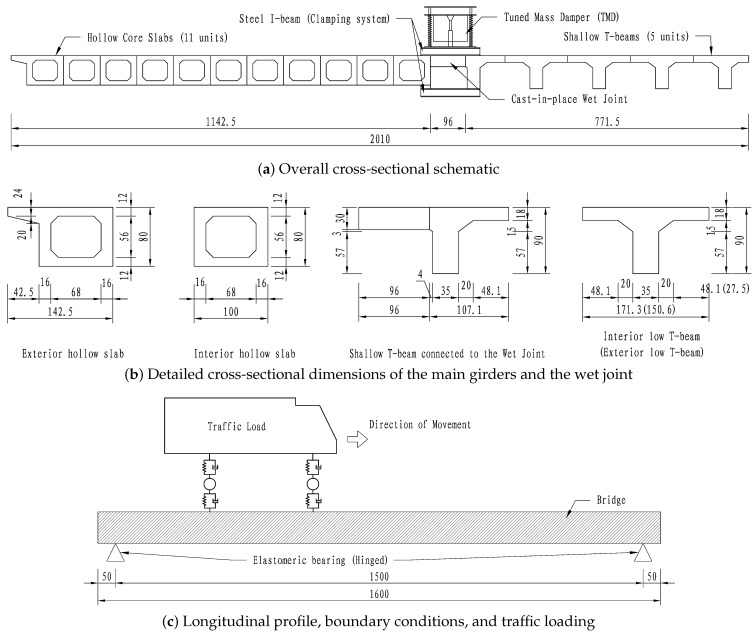
Geometric dimensions, boundary conditions, and loading schematic of the widened bridge model (unit: cm).

The widened bridge model investigated in this study employs a rigid connection between the old and new bridge sections. The main bridge, with a computational span of 16 m, consists of the existing bridge (hollow-core slabs), the new bridge (shallow T-beams), and the longitudinal joint between them. To clearly illustrate the analysis object, Figure 3 presents the detailed geometric dimensions, boundary conditions, and loading schematic.

- **Existing Bridge:** Composed of 11 prestressed concrete hollow-core slab beams connected by hinge joints. Each slab has a height of 80 cm, a width of 99 cm, and a center-to-center spacing of 100 cm (including a 1 cm hinge gap). The top and bottom slabs are both 12 cm thick, and the web is 16 cm thick.
- **New Bridge:** Composed of 5 prestressed concrete shallow T-beams connected by wet joints and diaphragms. Each beam has a height of 90 cm and a width of 120 cm. The top slab is 18 cm thick, and the web is 35 cm thick. Three diaphragms, each 35 cm thick, are located at the mid-span and at both supports.
- **Connection Joint:** The wet joint connecting the old and new sections has a width of 96 cm and a thickness of 30 cm.
- **Bridge Deck System:** The widened bridge deck accommodates a total of 5 traffic lanes. The pavement consists of a 10 cm thick C50 cast-in-place concrete layer and a 10 cm thick asphalt concrete layer.

Regarding the boundary conditions (Figure 3c), since the main girders are supported by elastomeric bearings, both ends are modeled as hinged in the longitudinal and vertical directions, which constrain the translational displacements in these directions while allowing rotation. For the loading conditions, the operational traffic loads are simulated as moving vehicle loads applied directly to the deck surface.

#### 3.2.3. Finite Element Modeling of the Bridge Structure

Based on the structural characteristics, the finite element model includes several components: the old hollow-core slabs, the new shallow T-beams, the old hinge joints, the new wet joints, the new diaphragms, the new–old connection joint, the approach slabs, and the pavement layers.

All components are modeled using SOLID45 solid elements. The element mesh size is determined by considering the support locations, the stability of the vehicle–bridge contact algorithm, and computational efficiency. Considering that the traffic-induced stresses in the wet joints and main girders are relatively small and remain strictly within the elastic range, a linear elastic constitutive model is adopted for all concrete materials in the FEM analysis. Regarding material properties, the new–old connection joint is modeled with BMF-based UHPC, while all other structural parts are modeled with C50 concrete (elastic modulus E = 3.45 $\times \;10^{4}$ MPa). The Poisson’s ratio for both concrete types is 0.20, and the density is 2600 kg/m^3^.

**Figure 4:**
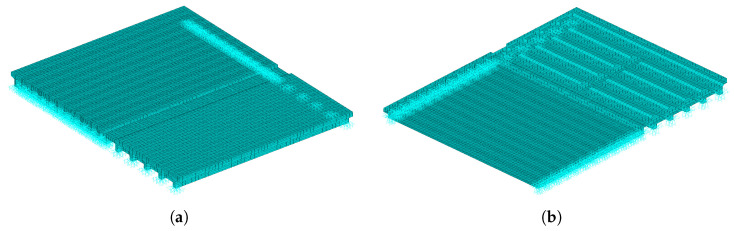
Finite element mesh model of the hollow-core slab and shallow T-beam widened bridge with boundary conditions. (**a**) Overall view. (**b**) Bottom-up view.

The finite element model of the widened bridge, composed of hollow-core slabs and shallow T-beams, is established. To clearly illustrate the numerical setup, the generated finite element mesh and the applied boundary conditions (hinged supports) are presented alongside the overall view and a bottom-up view of the model in Figure 4.

### 3.3. Road Surface Roughness and Its Simulation

Road surface roughness is the primary external excitation source that induces vibrations in the vehicle–bridge coupled system [20]. It is typically assumed to be a zero-mean Gaussian random process, and its statistical properties can be described by a Power Spectral Density (PSD) function. In this study, time-domain samples of road surface roughness are generated using the harmonic superposition method. This method is based on the PSD in the frequency domain and uses an Inverse Fast Fourier Transform (IFFT) to superimpose a series of cosine functions with random phases, thereby generating time-domain roughness data. According to the national standard GB/T 7031-2005 [21], the power spectral density of road surface roughness can be expressed as:

(4) $$G_{d}\left(n\right)=G_{d}\left(n_{0}\right){\left(\frac{n}{n_{0}}\right)}^{-W}$$

where:

$G_{d}\left(n\right)$ is the power spectral density (m^3^) at the spatial frequency *n* (m^−1^);
$G_{d}\left(n_{0}\right)$ is the road roughness coefficient at the reference spatial frequency $n_{0}$;
$n_{0}$ is the reference spatial frequency, typically taken as 0.1 m^−1^;
*W* is the frequency index, typically taken as 2;
*n* is the spatial frequency, defined as n=f/v, where *f* is the temporal frequency (Hz) and *v* is the vehicle speed (m/s).

For each discrete spatial frequency point $n_{k}$, the spectral amplitude of the road surface roughness is calculated as:

(5) $$A_{k}=\sqrt{2\cdot G_{d}\left(n_{k}\right)\cdot \Delta n}$$

where Δn is the spatial frequency sampling interval.

Through the Inverse Fast Fourier Transform (IFFT), the frequency-domain signal is converted into the time-domain road surface roughness elevation sample r(x):

(6) $$r\left(x_{j}\right)=\sum_{k=1}^{N}A_{k}\cos\left(2\pi n_{k}x_{j}+\phi_{k}\right)$$

where $\phi_{k}$ is a random phase angle uniformly distributed in the interval [0,2π].

**Figure 5:**
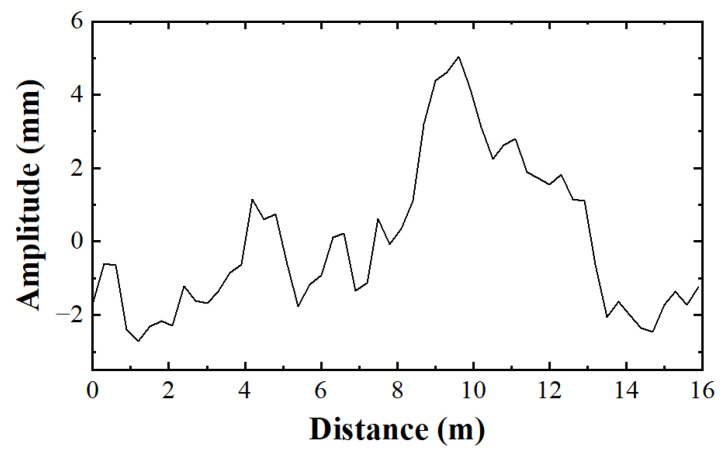
Generated road roughness profile for Grade A road condition according to GB/T 7031.

In this study, the selected effective spatial frequency range is from 0.01 m^−1^ to 3 m^−1^, ensuring that the vibration frequencies excited by the road roughness can cover the main natural frequencies of the vehicle systems at common speeds. Road surface roughness grades are classified according to the national standard GB/T 7031-2005, where Grade A represents a road roughness coefficient $G_{d}\left(n_{0}\right)$ between $8\times 10^{-6}$ and $32\times 10^{-6}$ m^2^/m^−1^. Considering that this study aims to analyze the dynamic response under good road conditions and that road roughness is not the core research variable, only Grade A road surface roughness is selected for the analysis. Samples for Grade A roughness were generated according to this standard (as shown in Figure 5) for use in the subsequent vehicle–bridge interaction analysis.

### 3.4. Point-to-Surface Contact Coupling Method

Traditional displacement coupling methods require the wheel application points to coincide exactly with the nodes of the bridge model. This often necessitates an extremely fine mesh for the bridge, which significantly increases model complexity and computational cost, making it particularly impractical for large-scale, complex bridge models. To overcome this limitation, this study adopts an improved point-to-surface contact coupling method.

**Figure 6:**
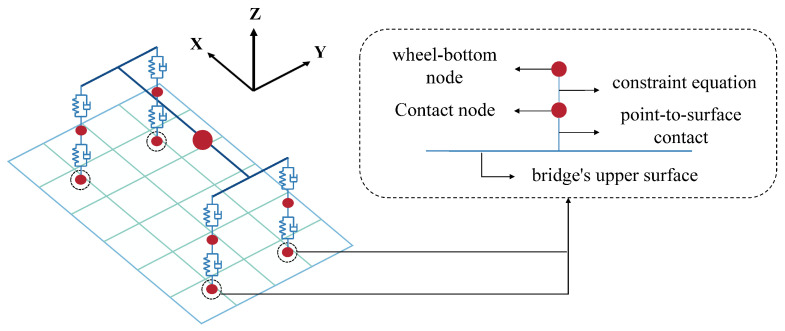
Schematic diagram of the improved point-to-surface contact coupling method.

This method ingeniously combines the advantages of the displacement contact method and constraint equations, specifically for solving vehicle–bridge interaction problems that consider road surface roughness. The core idea (as shown in Figure 6) is as follows: First, a point-to-surface contact pair is established between the wheel-bottom node (the point) and the bridge’s upper surface (the surface). This allows the wheel to move to any arbitrary position on the bridge deck without being restricted to node locations. Next, a constraint equation is used to link the degrees of freedom (DOFs) of the wheel with the DOFs of the contact point on the surface. Crucially, the road roughness value is introduced as a constant term in this equation, thereby achieving both displacement coordination and the application of the external excitation.

In this manner, the point-to-surface contact method not only solves the problem of non-coincident wheel–bridge nodes but also accurately simulates the excitation effect of road roughness on the vehicle–bridge system. Concurrently, the interaction forces between the wheel and the bridge are resolved through the contact algorithm, and the force equilibrium conditions are automatically satisfied. Therefore, this method effectively avoids the mesh refinement issues associated with traditional displacement coupling, providing an efficient and precise numerical implementation tool for dynamic analysis under complex operating conditions.

## 4. Calculation Results and Analysis

This chapter presents the results of the vehicle–bridge coupled dynamic analysis. First, the influence of curing age on the elastic modulus of the wet-joint concrete is elucidated along with the corresponding calculation methods. Subsequently, the key control indicators—vibration deformation (relative displacement) and vibration velocity—are defined. Finally, based on the established finite element model and coupling algorithm, the vibration responses of the bridge under different vehicle types, speeds, and concrete ages are systematically analyzed to evaluate their impact on the performance of the wet joint.

### 4.1. Parameter Definition of BMF-Based UHPC for Wet Joints

To accurately simulate the mechanical property evolution of BMF-based UHPC wet joints at early ages, macroscopic empirical formulas designed for traditional concrete can no longer accurately describe its characteristics. Based on the systematic research report on UHPC material properties published by the Federal Highway Administration (FHWA) (Graybeal, 2006) [22] and the closest particle packing theory, this paper redefines the mix proportion of the joint material and derives a time-varying elastic modulus calculation model for UHPC at different ages.

#### 4.1.1. Typical Mix Proportion Design and Microscopic Mechanism of BMF-Based UHPC

To ensure the joint material in the numerical simulation possesses sufficient engineering representativeness, this study deeply analyzes the mix proportion design logic of UHPC widely used in cast-in-place wet joints for bridge widening. Unlike traditional cementitious materials, the core of the UHPC mix design is based on the Modified Andreasen & Andersen (MAA) particle packing model. This system completely eliminates coarse aggregates and achieves extreme matrix densification by eliminating macroscopic pores and weak interfacial transition zones (ITZ) through the geometric compounding and chemical synergy of multi-scale powder materials [23].

Considering the on-site construction environment of bridge wet joints, this paper selects two highly representative cast-in-place UHPC mix proportions (as shown in Table 2). Mix I is a classic early-strength system pursuing ultimate strength; Mix II addresses the engineering challenge of cracking vulnerability in mass cast-in-place structures by introducing supplementary cementitious materials (SCMs), such as fly ash/slag, to replace part of the cement. While maintaining dense packing, it effectively reduces the hydration heat peak and early-age autogenous shrinkage risk during field casting.

#### 4.1.2. Early-Age Time-Varying Mechanical Property Model and Parameter Unification of BMF-Based UHPC

Although “Mix I (Classic Early-Strength)” and “Mix II (Cast-in-Place Low-Shrinkage)” listed in Table 2 differ in specific cementitious material compositions (e.g., Mix II introduces fly ash/slag), their core physical architectures strictly follow the closest particle packing model (MAA), and both incorporate a 2.0% volume fraction of basalt micro fibers (BMF). Compared to traditional steel fibers, BMF possesses a thermal expansion coefficient highly compatible with the concrete matrix and excellent corrosion resistance [24]. More importantly, under early-age vehicle-induced vibrations, the dense three-dimensional network formed by BMF effectively bridges microcracks and mitigates the ITZ degradation caused by particle displacement [25,26]. From the perspective of macroscopic mechanics and finite element equivalence, the addition of SCMs primarily serves to delay the early hydration heat peak and reduce autogenous shrinkage strain, but it does not alter the ultimate stiffness limit determined by the ultra-low water–binder ratio and multi-level dense skeleton. Therefore, at the level of structural response in numerical simulations, the macroscopic stiffness responses of these two mixes after curing are highly consistent, allowing for the adoption of the same time-varying constitutive equation and a unified reference elastic modulus for mechanical equivalent representation.

Considering that vehicle loads primarily disturb the matrix microstructure during the early window period of wet joint construction, a time-varying model capable of accurately capturing the extremely early stiffness evolution must be introduced. According to the systematic research report on UHPC material properties published by the FHWA (Graybeal, 2006) [22], the elastic modulus of UHPC exhibits a highly positive correlation with the square root of its compressive strength, with its classic prediction formula being:

(7) $$E=3840\sqrt{f_{c}}$$

To simulate the continuous dynamic changes from the initial setting of the paste to the entire hardening process, this paper introduces a time-varying development equation suitable for rapid-hardening and high-strength systems. The compressive strength $f_{c}\left(t\right)$ of UHPC at age *t* (days) is predicted as follows:

(8) $$f_{c}\left(t\right)=f_{c}\left(28\right)\cdot \exp\left(s\cdot \left[1-{\left(28/t\right)}^{0.5}\right]\right)$$

where $f_{c}\left(28\right)$ is the 28-day compressive strength; *s* is the hydration rate coefficient depending on the characteristics of the cementitious system, which is uniformly taken as s=0.2 for the cast-in-place UHPC joint system described in this paper.

Based on the characteristics of the high-strength matrix after sufficient hydration and densification, the compressive strength of UHPC in an ideal state can typically reach over 120 MPa. However, considering that the bridge widening wet joint is subjected to a field-cast curing environment, lacking standard laboratory or high-temperature steam curing conditions, and is limited by on-site temperature and humidity fluctuations, this paper conservatively sets the target ultimate compressive strength of the cast-in-place UHPC in the finite element model to $f_{c}^{\prime }=100$ MPa The calculated time-varying elastic modulus values of the wet joint material at key age nodes are presented in Table 3. These stiffness parameters, which are strictly bound to the time dimension (especially the sensitive period of the first 12 h), will be directly input into the vehicle–bridge coupled dynamic model to drive the solution of the nonlinear contact algorithm.

### 4.2. Determination of Control Indicators

#### 4.2.1. Cracking Control Indicators for Wet Joints

For newly cast concrete joints, traffic-induced vibrations may lead to early-age cracking, which subsequently compromises the long-term structural performance. Therefore, identifying appropriate critical vibration thresholds is essential for the evaluation and prevention of such cracks.

The Technical Specification for Repair and Connection of Bridge Concrete Structures under Uninterrupted Traffic (Guangdong Provincial Highway Society) [27] previously stipulated that during the interval between pouring and final setting, the vibration amplitude at the joint should not exceed 3 mm, with a frequency limit of 8 Hz. However, this unified limit possesses inherent limitations as it fails to account for variations in the geometric dimensions and mechanical characteristics of different wet joints.

Research by scholars such as Furr [28], Manning [29], Issa [30] and Kwan [31,32] has demonstrated that assessing the critical vibration state based on cracking performance is a more intuitive and reliable approach. Through extensive experimental studies on the effects of vibration on the performance of fresh concrete in wet joints, they proposed using the differential deflection (relative displacement) between the old and new bridge sections, or its corresponding vertical curvature, as the control indicators for structural cracking.

In this study, Kwan’s experimental results are adopted as the evaluation criteria for the impact of traffic-induced vibrations on the performance of concrete wet joints. Kwan categorized the cracking states into four levels—impending, slight, moderate, and severe—based on crack width. The corresponding critical vibration thresholds are summarized in Table 4.

The formula for joint curvature is:

(9) $$\rho =\frac{9\Delta }{10L^{2}d}$$

where:

Δ is the relative displacement (deflection difference) between the joint ends;
*L* is the width of the joint (960 mm);
*d* is the thickness of the joint (300 mm).

For the rigid joint in this study, the critical curvature at the point of “impending cracking” is $18\times 10^{-3}$ m^−1^. Substituting the joint dimensions, the critical relative displacement is calculated as 5.5 mm. Thus, 5.5 mm is set as the vertical relative displacement limit.

#### 4.2.2. Vibration Velocity Control Indicators

Regarding the vibration limit for newly cast UHPC, it should not be confined solely to the macroscopic stress level. As analyzed in the previous microscopic mechanism section, high-energy vibration will destroy the nano-mechanical framework of the UHPC interface and disrupt the directional arrangement of steel fibers. Although some international codes (such as the UK DMRB standard [33]) specify a limit of 5 mm/s for ordinary continuous rigid-frame bridges, considering the extremely high cost of UHPC and its stringent requirements for early-age steel-matrix interfacial transition zone (ITZ) denseness, a more conservative control strategy must be adopted.

Research by Sungnam Hong [34] and meso-scale tests by numerous scholars [35,36] indicate that strictly limiting the peak particle velocity (PPV) to below 3 mm/s during the first 6 to 12 h after concrete casting (i.e., the critical molding period from initial to final setting) can effectively prevent interfacial slip caused by bleeding and particle rearrangement inside the matrix, thereby creating a “quasi-static” curing environment. Therefore, this paper adopts 3 mm/s as the critical vibration velocity control threshold for the microstructural protection of UHPC wet joints.

#### 4.2.3. Temporary Connection Measures

To ensure that traffic remains operational during the construction of the cast-in-place wet joints while protecting the fresh concrete from harmful vibrations, the following engineering measures were implemented:

1. **Temporary Steel Connecting Clamps:** These were installed between the existing and new girders to facilitate joint load-sharing under vehicle loads. This measure ensures the continuity and coordination of deformations between the old and new hollow-core slabs.
2. **Anchorage via Self-tapping Bolts:** Self-tapping bolts were implanted into the edge beams of the existing bridge. After the installation of the new bridge girders, deck pavement, and outer guardrails, the original crash barriers were cut and removed. Subsequently, the self-tapping bolts were welded to the reinforcement bars embedded in the new bridge.
3. **Monolithic Pouring:** The bridge deck pavement (spanning approximately 2 m transversely across the connection) and the wet joint concrete were poured simultaneously. Furthermore, the reinforcement mesh within this pavement area was strengthened.

These measures are designed to restrict the differential deflection and vibration levels experienced by the wet-joint concrete during its strength-development phase, ensuring that the casting and curing quality meet the specified requirements.

### 4.3. Calculation Results

**Figure 7:**
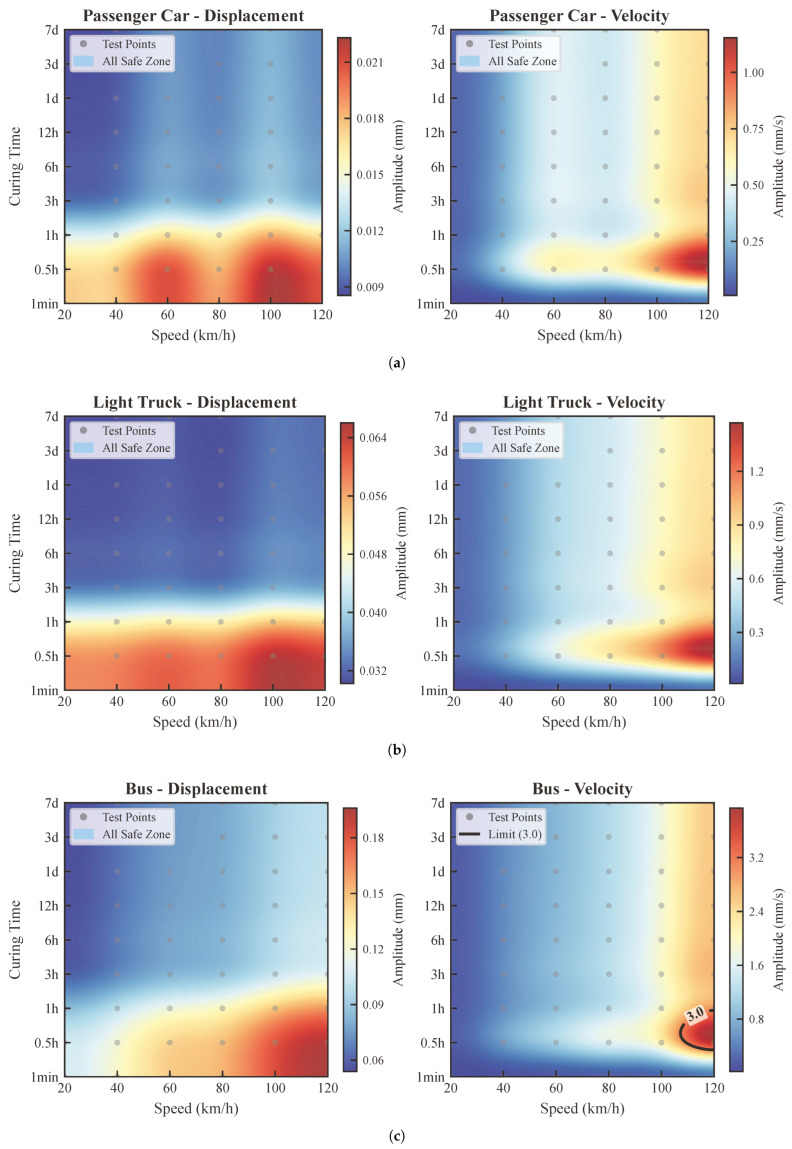

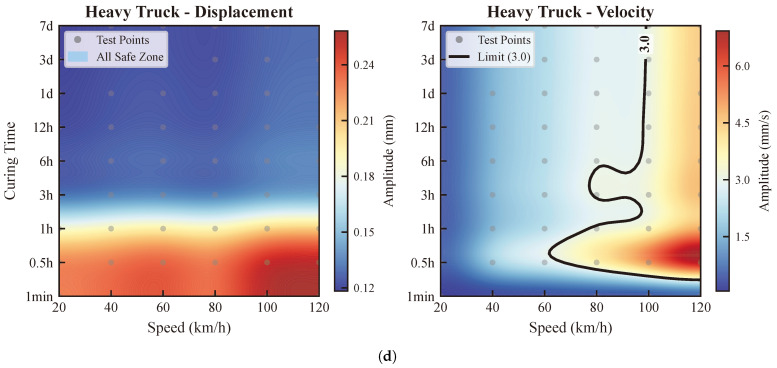
Contour maps of the maximum vertical relative displacement and peak velocity at the mid-span wet joint under different vehicle speeds and curing ages. (**a**) Passenger Car. (**b**) Light-duty Truck; (**c**) Bus; (**d**) Heavy-duty Truck.

This section analyzes the vibration response of the rigid wet joint as four types of vehicles—passenger car, light-duty truck, bus, and heavy-duty truck—traverse the widened bridge at various speeds and concrete curing ages (as shown in Figure 7). Each figure consists of two subplots: the left subplot displays the vertical relative displacement of the joint, while the right subplot shows the peak vibration velocity. The analysis covers six vehicle speeds ranging from 20 km/h to 120 km/h and nine distinct concrete ages.

#### 4.3.1. Vibration Deformation (Vertical Relative Displacement) Analysis

Based on the relative displacement plots in Figure 7, it is observed that under all tested vehicle speeds and concrete ages, the vertical relative displacements induced by the four vehicle types remain below the critical limit of 5.5 mm. This result indicates that the implementation of temporary steel connecting clamps effectively restricts the relative movement between the old and new bridge sections. Consequently, it is ensured that the wet-joint concrete will not undergo cracking due to excessive deformation during its early curing stages.

#### 4.3.2. Vibration Velocity Analysis

Based on the velocity contour plots shown in Figure 7 (with 3 mm/s defined as the critical threshold), the vibration characteristics induced by different vehicle types under varying speeds and curing ages were analyzed. The specific conclusions are as follows:

According to the peak velocity plots (where the black dashed line represents the 3 mm/s limit), the following conclusions can be drawn:

- **Passenger Cars and Light-duty Trucks:** The vibration velocities induced by lightduty vehicles remain consistently below the 3 mm/s limit across the entire speed range (0–120 km/h). This indicates that the impact of such vehicles on the early-age performance of the concrete is negligible, and no specific traffic restrictions are required.
- **Buses:** The vibration induced by buses shows a clear correlation with the curing age. The data indicates that the vibration velocity drops below 3 mm/s after 1 h of curing. This implies that the restriction window for buses is relatively short; it is recommended to restrict their passage only during the first hour after the initial setting of the concrete.
- **Heavy-duty Trucks:** Heavy trucks exert the most significant dynamic impact on the joint concrete. Within the first 12 h of curing, the generated vibration velocities generally exceed the safe threshold of 3 mm/s. This highlights the first 12 h as a critical “curing window” for preventing early-age damage. Consequently, it is strongly recommended to strictly prohibit heavy truck traffic during this period and allow access only after the concrete has gained sufficient strength (i.e., after 12 h).

#### 4.3.3. Comprehensive Evaluation

Overall, the installation of temporary steel connecting clamps plays a significant role in controlling the relative displacement between the old and new bridge sections. However, regarding vibration velocity, heavy vehicles—particularly trucks—can still cause the joint to exceed safety limits during the early curing stages (within 12 h). Therefore, in practical engineering, heavy truck traffic should be strictly limited based on the traffic load type and the curing age of the concrete. Alternatively, further vibration mitigation strategies (such as the TMD system proposed in Section 5) should be adopted to safeguard construction quality and long-term durability.

## 5. Vibration Control Based on Tuned Mass Dampers (TMD)

### 5.1. Parameter Design of the TMD System

The Tuned Mass Damper (TMD) is a passive control device that suppresses the vibration response of a primary structure through precise frequency tuning of its dynamic subsystem, utilizing resonance energy absorption and damping dissipation mechanisms. Considering engineering simplicity and maintenance costs, this study employs a single-unit TMD to control the critical vibration modes of the bridge.

#### 5.1.1. Dynamic Model and Optimization Criteria

For the parameter optimization of the TMD system, Den Hartog’s classical tuning theory [37] is adopted as the fundamental criterion. A critical constraint in this optimization process is the mass ratio (μ), defined as the ratio of the TMD mass to the primary structure’s effective modal mass. To prevent excessive additional dead load on the newly widened structure while ensuring sufficient energy dissipation, μ was strictly evaluated within a typical engineering range of [0.01, 0.05]. Based on this defined domain, the objective functions for the optimal frequency ratio ($\alpha_{opt}$) and the optimal damping ratio ($\xi_{opt}$) are established to minimize the dynamic response of the bridge under moving loads.

#### 5.1.2. Parameter Determination Procedure

Based on the dynamic characteristics of the bridge, the design parameters of the TMD—namely mass ($m_{d}$), stiffness ($k_{d}$), and damping coefficient ($c_{d}$)—are determined according to the following mechanical relationships:

1. **Mass Ratio Determination:** The mass ratio μ is defined as the ratio of the TMD mass $m_{d}$ to the first-order effective modal mass *M* of the primary structure:
  (10) $$\mu =\frac{m_{d}}{M}$$
2. **Optimal Frequency Ratio ($\alpha_{opt}$) and Optimal Frequency ($\omega_{d}$):**
  (11) $$\alpha_{opt}=\frac{1}{1+\mu }$$
  (12) $$\omega_{d}=\alpha_{opt}\cdot \omega_{b}$$
  where $\omega_{b}$ is the target natural circular frequency of the bridge.
3. **Optimal Damping Ratio ($\xi_{opt}$):**
  (13) $$\xi_{opt}=\sqrt{\frac{3\mu }{8(1+\mu )}}$$
4. **Calculation of Physical Component Parameters:** Based on the determined mass and frequency, the stiffness and damping coefficients are derived as:
  (14) $$k_{d}=m_{d}\cdot \omega_{d}^{2}$$
  (15) $$c_{d}=2m_{d}\cdot \omega_{d}\cdot \xi_{opt}$$

#### 5.1.3. Detailed Parameter Design

**Figure 8:**
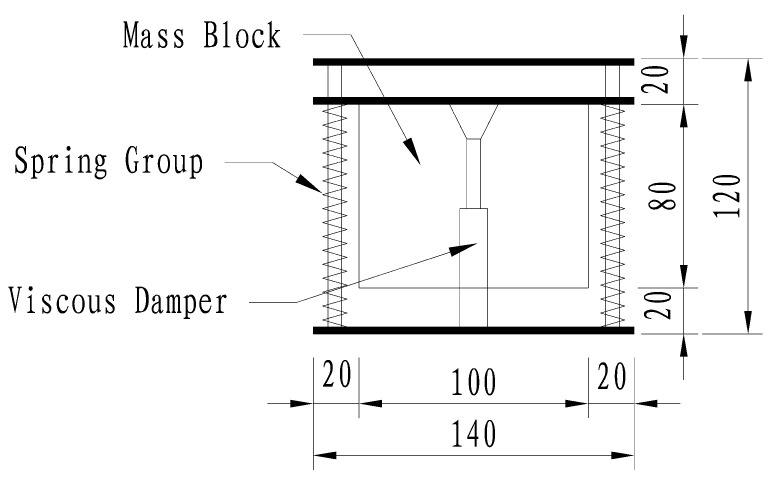
Conceptual schematic and physical dimensions (unit: cm) of the single-unit TMD device, illustrating the mass block, spring group, and viscous damper, as referenced by Table 5.

According to the aforementioned dynamic analysis, the bridge exhibits a first natural circular frequency of $\omega_{b}=69.1$ rad/s and an effective modal mass of M=311,368 kg. The selection of the mass ratio μ represents a critical trade-off between vibration suppression efficiency and structural weight constraints. While a higher mass ratio generally enhances energy dissipation, it also introduces significant additional dead load and requires larger installation space. Conversely, a mass ratio below 0.01 may result in insufficient control over the peak dynamic responses. After a comparative evaluation, a mass ratio of μ=0.02 was adopted. This value ensures substantial reduction in bridge vibrations while maintaining the additional mass at a manageable level (approx. 6227 kg), thereby optimizing the cost-effectiveness and structural safety of the intervention. As shown in Figure 3, the TMD units are installed directly on the temporary steel clamps (I-beams) spanning the new and existing girders. By utilizing these clamps as a rigid support platform, this arrangement eliminates the need for auxiliary fixtures and places the dampers close to the vibration-sensitive wet joints without obstructing concrete casting, thus balancing construction feasibility with functional performance. A conceptual schematic of the single-unit TMD device, highlighting its key physical dimensions and internal components based on the design parameters, is presented in Figure 8. Detailed design parameters are presented in Table 5.

### 5.2. Analysis of Vibration Reduction Effectiveness

**Figure 9:**
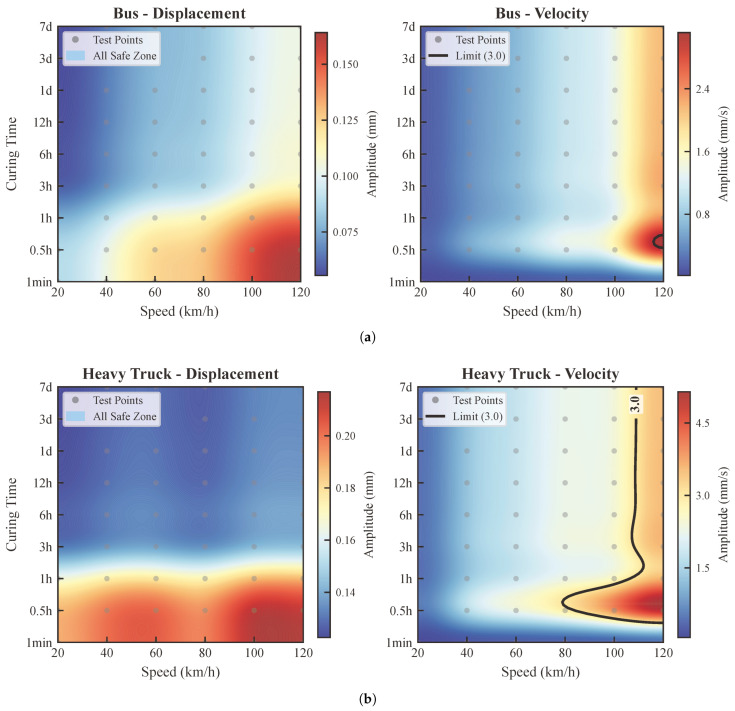
Contour maps of the maximum vertical relative displacement and peak velocity at the mid-span wet joint with the TMD system under different vehicle speeds and curing ages. (**a**) Bus. (**b**) Heavy-duty Truck.

To evaluate the effectiveness of the designed TMD system, this study focuses on its ability to control the vibration response for buses and heavy trucks, which were previously found to exceed the vibration velocity limits. Without the TMD, these heavy vehicles induced vibration velocities exceeding the 3 mm/s control threshold under several operating conditions. Upon configuring the TMD system within the structure (as shown in Figure 9), the vibration velocity is significantly suppressed. For buses, the vibration velocity satisfies the limit requirements across all speeds up to 120 km/h, effectively resolving the over-limit issues during the early curing stages. For heavy trucks, the vibration velocity is successfully maintained below 3 mm/s for speeds less than or equal to 80 km/h.

Comprehensive evaluation indicates that the TMD system is highly effective in controlling the vibration velocity induced by heavy vehicles, serving as a robust measure to ensure the quality of wet joints during construction. This system allows for the safe passage of buses under all conditions, significantly improving early-stage traffic capacity. For heavy trucks, although the reduction effect has certain limitations at high speeds (>80 km/h), the damage risk to early-age concrete can still be effectively mitigated when combined with traffic control measures (speed limits ≤ 80 km/h), demonstrating the engineering value of the proposed scheme.

### 5.3. Recommendations for Vibration Management Measures

Based on the research findings, this paper proposes a differentiated vibration control strategy based on traffic composition characteristics, aimed at balancing structural safety, cost-effectiveness, and traffic demands.

Low-impact Zones: For road sections dominated by passenger cars and light trucks with a low proportion of heavy vehicles, the vibration response typically remains within the 3 mm/s safety limit. In such cases, the installation of a TMD system is not recommended; conventional construction management is sufficient. This approach significantly reduces the costs associated with TMD design, fabrication, installation, and maintenance while simplifying construction organization.

High-impact Zones: For sections where buses and heavy trucks predominate, a synergistic control strategy combining passive control and active management must be implemented. First, an optimized TMD system should be installed as the primary technical intervention to suppress bus vibrations and keep truck vibrations within safe ranges under speed restrictions. Second, active traffic management should be enforced during the critical curing period (e.g., the first 7 days post-pouring), limiting heavy truck speeds to 80 km/h or less. This dual-layer strategy leverages the efficacy of passive devices while utilizing administrative measures to compensate for limitations under extreme operating conditions.

Finally, to ensure design validity and account for on-site uncertainties, real-time vibration monitoring of the wet joints is strongly recommended during the construction and initial traffic opening phases. By installing vibration sensors to collect Peak Particle Velocity (PPV) data, a closed-loop “design-monitoring-feedback-adjustment” management model can be established. If measured data consistently remain below the 3 mm/s threshold, restrictions may be gradually relaxed; conversely, if a risk of exceeding the limit is detected, stricter measures should be immediately enforced to guarantee the integrity of the engineering project.

## 6. Conclusions

This study investigated the early-age vibration damage of wet joints in bridge widening projects under uninterrupted traffic, specifically focusing on the widening of hollow-core slab bridges with shallow T-beams. By establishing a refined vehicle–bridge interaction (VBI) dynamic model and performing a systematic parametric analysis, the following conclusions were drawn:

Vibration Response Patterns: In the absence of additional vibration reduction measures, although the temporary steel connection fixtures can effectively control the relative displacement between the new and old bridges within the critical value of 5.5 mm, the peak particle velocity (PPV) generated by heavy vehicles (buses and trucks) extensively exceeds the safety threshold of 3 mm/s during the early stage. Under this high-energy vibration, microstructural degradation and random orientation of steel fibers in the BMF-based UHPC interfacial transition zone (ITZ) are highly likely to be triggered, constituting the core microscopic factor that poses hidden dangers to the long-term durability of wet joints.

Efficacy of the TMD System: The single-unit Tuned Mass Damper (TMD), optimized for the first-order natural frequency of the bridge, demonstrates significant vibration suppression capabilities. Following the configuration of the TMD system, the vibration response induced by buses is reduced below the safety threshold across the entire speed range (≤120 km/h ); furthermore, vibrations induced by heavy-duty trucks are effectively controlled at speeds of 80 km/h and below.

Differentiated Management Strategy: A comprehensive framework combining “technical interventions” and “administrative management” is proposed. Specifically, the “technical interventions” primarily refer to the deployment of the optimized TMD system (μ=0.02) utilizing temporary steel clamps. This structural measure effectively suppresses the traffic-induced vibrations transmitted to the UHPC wet joints during their critical early-age curing phase, without requiring permanent structural alterations to the main girders. The ”administrative management” encompasses temporary traffic regulation strategies enforced on the existing bridge during construction, such as restricting the access of heavy-duty trucks and scheduling concrete casting and initial curing during off-peak hours (e.g., nighttime). Based on this synergy, for road sections with a high volume of heavy traffic, a strategy of “passive TMD mitigation + truck speed limits (≤80 km/h)” should be mandated. For sections dominated by light-duty vehicles, conventional construction monitoring may be adopted to optimize economic costs.

Closed-loop Management Model: To address uncertainties in the construction environment and fluctuating traffic flows, the implementation of a real-time vibration monitoring system based on sensor networks is recommended. This establishes a “design-monitoring-feedback-adjustment” dynamic management loop, ensuring the construction quality and long-term durability of the wet joints.

In summary, the findings of this research clarify the impact mechanism of traffic-induced vibrations on widened bridge joints and provide a practical, integrated solution. This study offers scientific guidance for balancing construction quality with traffic efficiency in similar bridge expansion projects.

## Figures and Tables

**Table 1:** Main properties and dimensions of vehicle.

Parameter	Symbol	Unit	Cars	L.Trucks	Buses	H.Trucks
Mass of the rigid body	$M_{vr}$	kg	1280	6000	12,190	2276.5/45,245
Pitching moment of inertia	$I_{yv}$	kg·m^2^	2000	34,000	79,000	20,196/285,990
Mass of axle block	$M_{aj}$	kg	200/160	800/700	340/525	700/1000/1000/800/800
Upper vertical spring stiffness	$K_{uj}$	$10^{3}$ N/m	310/310	200/225	126/255	300/1000/1000/1250/1250
Upper vertical damping coefficient	$C_{uj}$	$10^{2}$ N·s/m	62/20	39/27	92/183	100/530/530/530/530
Lower vertical spring stiffness	$K_{lj}$	$10^{3}$ N/m	310/310	363/422	750/1500	1500/3000/3000/3000/3000
Lower vertical damping coefficient	$C_{lj}$	$10^{2}$ N·s/m	6/6	30/30	2 0/20	30/30/30/30/30
Dimensional parameters	$L_{j}$	m	1.7/1.4	2.6/3.0	3.4/1.8	1.0/2.0/1.4/3.8/3.2/1.4/2.7/4.5

**Table 2:** Two typical cast-in-place UHPC mix proportions for bridge wet joint engineering (Unit: kg/m^3^).

Component Category	Specific Material	Mix I (Classic Early-Strength)	Mix II (Cast-in-Place Low-Shrinkage)
Cementitious Materials	P.O 52.5 Portland Cement	712	650
	Silica Fume	231	150
	Fly Ash/Slag	-	220
Fine Aggregate	Quartz Sand	1020	1050
Powder Micro-fillers	Ground Quartz	211	180
Admixture	Superplasticizer (SP)	30.7	35
Reinforcement Phase	basalt micro fibers (BMF)	53 (2.0 Vol%)	53 (2.0 Vol%)
Water	Mixing Water	109	160

**Table 3:** Elastic modulus values of joint concrete at different ages.

Age	1 min	0.5 h	1 h	3 h	6 h	12 h	1 d	3 d	7 d	28 d
$E_{c}$ (×10^4^ MPa)	8.08 × 10^9^	0.11	0.32	0.95	1.47	2.01	2.50	3.13	3.47	3.84

**Table 4:** Critical vibration values for concrete cracking.

Cracking State	Impending	Slight	Moderate	Severe
Crack Width (mm)	-	0.1	0.2	>0.3
Critical Curvature ($\times 10^{-3}\;m^{-1}$)	18	27	36	45

**Table 5:** Design parameters of the TMD system.

Parameter	Symbol	Value	Unit
Effective mass ratio	μ	0.02	-
TMD mass	$m_{d}$	6227	kg
Optimal frequency ratio	$\alpha_{opt}$	0.9804	-
Tuned circular frequency	$\omega_{d}$	67.76	rad/s
Optimal damping ratio	$\xi_{opt}$	0.0857	-
Stiffness coefficient	$k_{d}$	28592	kN/m
Damping coefficient	$c_{d}$	72.366	kN·s/m

## Data Availability

The original contributions presented in this study are included in the article. Further inquiries can be directed to the corresponding author.
